# Hypoxia-induced expression of cellular prion protein improves the therapeutic potential of mesenchymal stem cells

**DOI:** 10.1038/cddis.2016.310

**Published:** 2016-10-06

**Authors:** Yong-Seok Han, Jun Hee Lee, Yeo Min Yoon, Chul Won Yun, Hyunjin Noh, Sang Hun Lee

**Affiliations:** 1 Medical Science Research Institute, Soonchunhyang University Seoul Hospital, Seoul, Republic of Korea; 2 Department of Pharmacology and Toxicology, University of Alabama at Birmingham School of Medicine, Baltimore, AL 35294, USA; 3 Department of Internal Medicine, Soonchunhyang University, Seoul, Republic of Korea; 4 Hyonam Kidney Laboratory, Soonchunhyang University, Seoul, Republic of Korea; 5 Departments of Biochemistry, Soonchunhyang University College of Medicine, Cheonan 330-930, Republic of Korea

## Abstract

Mesenchymal stem cells (MSCs) are ‘adult' multipotent cells that promote regeneration of injured tissues *in vivo*. However, differences in oxygenation levels between normoxic culture conditions (21% oxygen) and both the MSC niche (2–8% oxygen) and ischemic injury-induced oxidative stress conditions *in vivo* have resulted in low efficacy of MSC therapies in both pre-clinical and clinical studies. To address this issue, we examined the effectiveness of hypoxia preconditioning (2% oxygen) for enhancing the bioactivity and tissue-regenerative potential of adipose-derived MSCs. Hypoxia preconditioning enhanced the proliferative potential of MSCs by promoting the expression of normal cellular prion protein (PrP^C^). In particular, hypoxia preconditioning-mediated MSC proliferation was regulated by PrP^C^-dependent JAK2 and STAT3 activation. In addition, hypoxia preconditioning-induced PrP^C^ regulated superoxide dismutase and catalase activity, and inhibited oxidative stress-induced apoptosis via inactivation of cleaved caspase-3. In a murine hindlimb ischemia model, hypoxia preconditioning enhanced the survival and proliferation of transplanted MSCs, ultimately resulting in improved functional recovery of the ischemic tissue, including the ratio of blood flow perfusion, limb salvage, and neovascularization. These results suggest that Hypo-MSC offer a therapeutic strategy for accelerated neovasculogenesis in ischemic diseases, and that PrP^C^ comprises a potential target for MSC-based therapies.

Human mesenchymal stem cells (MSCs) have numerous potential applications for regenerative medicine-based therapies for diseases such as cardiovascular disease, ischemic stroke, peripheral artery disease, spinal cord injury, liver disease, and anemia. However, various pathophysiological conditions, including ischemia, inflammation, and low nutrient levels, have inhibited the efficacy of MSC-based therapies.^1^ In particular, ischemia-mediated oxidative stress leads to the production of reactive oxygen species (ROS), resulting in DNA damage, cell apoptosis and death, and end-organ tissue damage.^2^ To address the low efficacy of transplanted MSCs, several studies have performed genetic modification of MSCs; however, the clinical application of this approach is limited owing to unexpected adverse effects, such as toxicity, immunogenicity, and oncogenicity.^3, 4^ Therefore, it is pertinent to search for novel approaches to promote transplanted cell survival to foster the success of stem cell-based therapies for ischemic diseases.

Ischemic tissues induce severe hypoxic conditions (O_2_ concentrations <0.1%),^5^ which often lead to low survival, inferior differentiation, and death of grafted cells.^6, 7^ Notably, however, previous studies demonstrated that hypoxia-induced apoptosis can be prevented in certain cell types by preconditioning the cells, via exposure to less severe hypoxic conditions (1–3% O_2_), for a period of time prior to exposing them to the severe ischemia at the injury site.^8, 9, 10^ Cultivation under hypoxic conditions (1–3% O_2_) may also be beneficial for MSCs, as this oxygen tension is similar to that present at the physiologic niche for MSCs in the bone marrow (2–7% O_2_). Although hypoxia preconditioning induced the upregulation of hypoxia-inducible factor-1 alpha (HIF-1*α*), CXCR4, c-Met, and/or TWIST, it is unclear how this treatment affects self-renewal, differentiation, and survival of various stem/progenitor cells in ischemic injured tissues.

The normal cellular isoform of prion protein (PrP^C^) is a highly conserved glycoprotein that is monomeric in structure, protease-sensitive, and tethered to the cell membrane by a glycosyl phosphatidyl inositol anchor.^11^ Meanwhile, the abnormal form of PrP is commonly multimeric, aggregated, and protease-resistant, and is associated with neurotoxicity and neurodegeneration.^12^ Although several studies investigated the molecular mechanisms underlying the role of the abnormal form of PrP in neuronal diseases, understanding the physiological role of PrP^C^ remains a challenge due to its numerous functions^13, 14^ and its roles in cellular processes such as proliferation, self-renewal, and differentiation.^15, 16, 17^ In addition, a recent study indicated that cellular prion protein enhances neuronal survival and angioneurogenesis after post-ischemic injury.^18^ However, it is unknown whether MSCs express PrP^C^ or whether PrP^C^ affects MSCs bioactivity, including survival and proliferation, under ischemic conditions.

In this study, we investigated whether cultivation under hypoxic conditions could be utilized to enhance the efficacy of MSCs for ischemic therapy relative to normoxic culturing. We first attempted to establish a general protocol for achieving increased MSC proliferation under hypoxic culture conditions by determining the effects of hypoxic preconditioning on MSC proliferative potential. We subsequently evaluated the ability of preconditioned cells to promote therapeutic vasculogenesis in ischemic limbs. Moreover, we investigated the mechanism by which PrP^C^ regulation affects the proliferation and survival of MSCs subjected to hypoxic preconditioning.

## Results

### Characterization of MSC differentiation and assessment of MSC proliferation under hypoxic culture conditions

**Figure 1:**
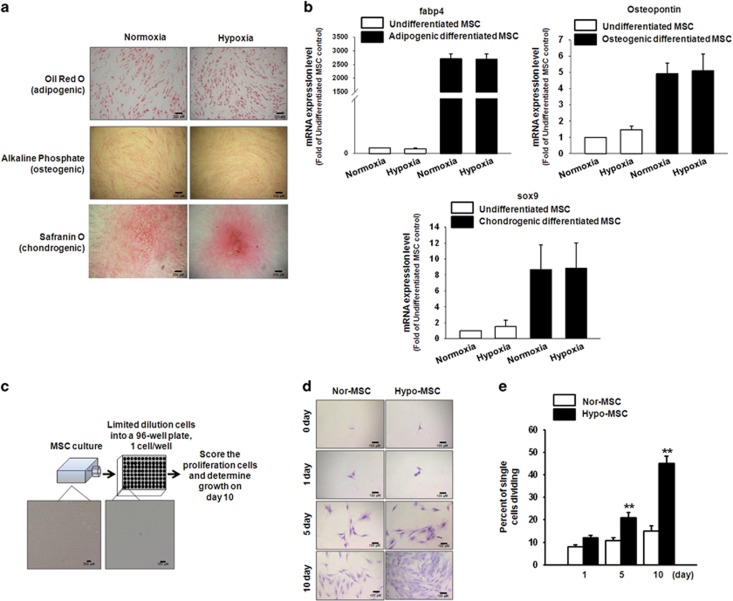
Effects of hypoxia preconditioning on the proliferation of mesenchymal stem cells (MSCs). (**a**) MSCs were differentiated into adipocytes, osteocytes, and chondrocytes under normoxic or hypoxic conditions *in vitro*. Adipogenic, osteogenic, and chondrogenic differentiation was examined by oil red O, alkaline phosphatase, and safranin O staining, respectively. Scale bar=200 *μ*m. (**b**) Adipogenic, osteogenic, and chondrogenic differentiation was examined by fabp2, osteopontin, and sox9 gene expression using quantitative real-time PCR. (**c**) Schematic of single-cell assays using MSCs. Scale bar=200 *μ*m. (**d**) Normoxic MSCs (Nor-MSC) and hypoxia-preconditioned MSCs (Hypo-MSC) were grown in single-cell cultures for 0, 5, and 10 days, stained with Giemsa (Scale bar=100 *μ*m), and (**e**) the percentage of single MSCs undergoing at least one cell division after 1, 5, and 10 days of culture. Values represent means±S.E.M. **P<0.01 *versus* Nor-MSC

There was no significant difference in MSC differentiation when cultivated under normoxic and hypoxic (2% O_2_) conditions, as determined by oil red O (adipogenesis), alkaline phosphate (osteogenesis), and safranin O (chondrogenesis) staining analyses, and fabp4 gene (adipogenesis), osteopontin gene (osteogenesis), and sox9 gene (chondrogenesis) expression did not change significantly (Figure 1a and b). To investigate their proliferative potential under hypoxic conditions, MSCs were evaluated using a single-cell expansion assay (Figure 1c). Notably, hypoxia-preconditioned MSCs (Hypo-MSC) exhibited significantly greater levels of expansion than MSCs cultivated under normoxic conditions (Nor-MSCs; Figure 1d and e). These data suggested that hypoxia preconditioning enhances MSC proliferation.

### Hypoxia preconditioning induces HIF-1*α*-mediated PrP^C^ expression

**Figure 2:**
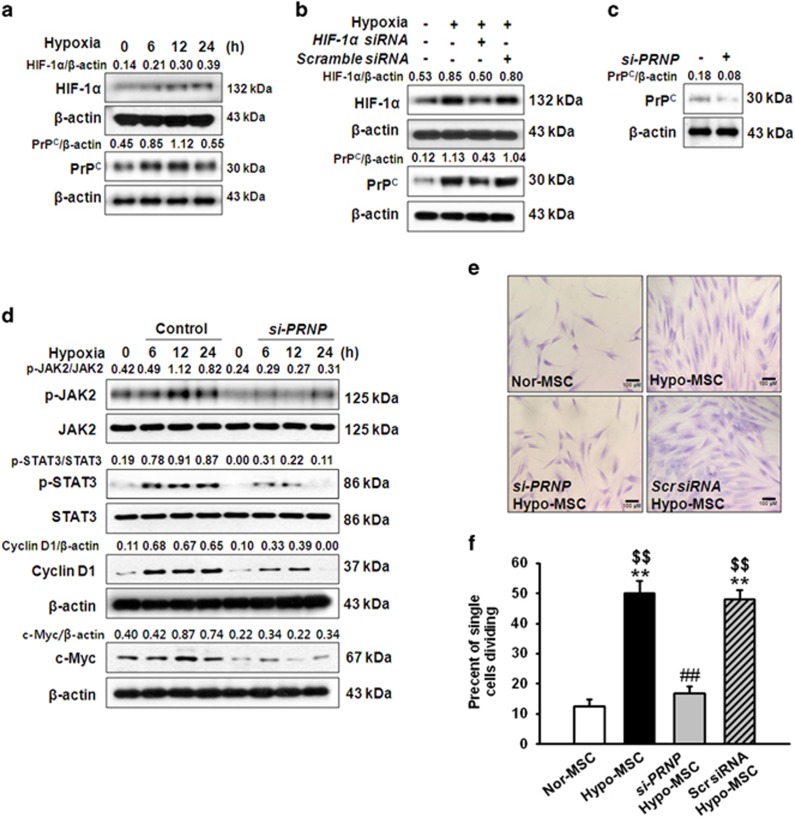
Hypoxia preconditioning increases cell proliferation through HIF-1*α*-mediated expression of cellular prion protein (PrP^C^). (**a**) Western blot analysis of HIF-1*α* and PrP^C^ expression in mesenchymal stem cells (MSCs) subjected to hypoxia preconditioning for 0, 6, 12, and 24 h. (**b**) Western blot analysis of HIF-1*α* and PrP^C^ expression in MSCs subjected to hypoxia preconditioning for 12 h and pretreated with HIF-1*α*-specific siRNA (si-HIF-1*α*). (**c**) Western blot analysis of PrP^C^ expression in MSCs pretreated with PrP^C^-specific siRNA (si-PRNP). (**d**) Western blot analysis of JAK2 and STAT3 phosphorylation, and cyclin D1 and c-Myc expression in MSCs and MSCs treated with PrP^C^-specific siRNA after hypoxia preconditioning for 0, 6, 12, and 24 h. (**e**) Single-cell cultures of normoxic MSCs (Nor-MSC), hypoxia-preconditioned MSCs (Hypo-MSC), hypoxia-preconditioned MSCs transfected with PrP^C^-specific siRNA (si-PRNP Hypo-MSC), and hypoxia-preconditioned MSCs transfected with scrambled siRNA (Scr siRNA Hypo-MSC) were stained with Giemsa after 10 days of cultivation. Scale bar=100 *μ*m. (**f**) Percentage of single MSCs undergoing at least one cell division after 10 days of culture. Values represent means±S.E.M. **P<0.01 *versus* Nor-MSC; ^##^*P*<0.01 *versus* Hypo-MSC; ^$$^*P*<0.01 *versus* si-PRNP Hypo-MSC

To explore whether hypoxia preconditioning induces PrP^C^ expression in MSCs, we evaluated the expression levels of HIF-1*α* and PrP^C^ in hypoxia-preconditioned cells by western blot analysis. Hypoxia preconditioning resulted in a time-dependent increase in HIF-1*α* expression (Figure 2a). Meanwhile, maximal levels of PrP^C^ expression were observed after hypoxic stimulation for 12 h (Figure 2a). Notably, however, this hypoxia-induced expression of PrP^C^ was attenuated by treatment of MSCs with HIF-1*α*-specific siRNAs (Figure 2b). These findings suggest that hypoxia preconditioning promotes the expression of PrP^C^ via hypoxia-mediated increases in HIF-1*α* expression.

### PrP^C^ regulates hMSC proliferation via the JAK2/STAT3 pathway

A previous study demonstrated that hypoxia preconditioning enhances cell proliferation through the JAK2/STAT3 signaling pathway.^19^ To confirm whether hypoxic conditions promote MSC proliferation through the JAK2/STAT3 pathway, hypoxia-induced phosphorylation of JAK2 and STAT3 were investigated by western blot analysis. Although hypoxia preconditioning for 12 h resulted in increased JAK2 and STAT3 phosphorylation, these effects were attenuated by treatment of MSCs with PrP^C^-specific siRNA molecules (Figure 2c and d). Likewise, we observed increased expression of cyclin D1 and c-Myc, which are encoded by STAT3 downstream genes, after hypoxic stimulation for 12 h, and decreased expression of these proteins upon siRNA-mediated knockdown of PrP^C^ expression (Figure 2d).

To explore the role of enhanced PrP^C^ expression on the proliferative potential of MSCs under hypoxic conditions, single-cell expansion assays were performed (Figure 2e). Notably, the increase in proliferation observed in Hypo-MSC, compared with that of MSCs cultivated under normoxic conditions, was inhibited by siRNA-mediated PrP^C^ knockdown (Figure 2f). Together, these results suggest that hypoxia preconditioning enhances MSC proliferation potential through PrP^C^-mediated activation of JAK2/STAT3 pathway.

### Hypoxia preconditioning-mediated increases in PrP^C^ expression provide a protective effect against oxidative stress-induced apoptosis in MSCs

**Figure 3:**
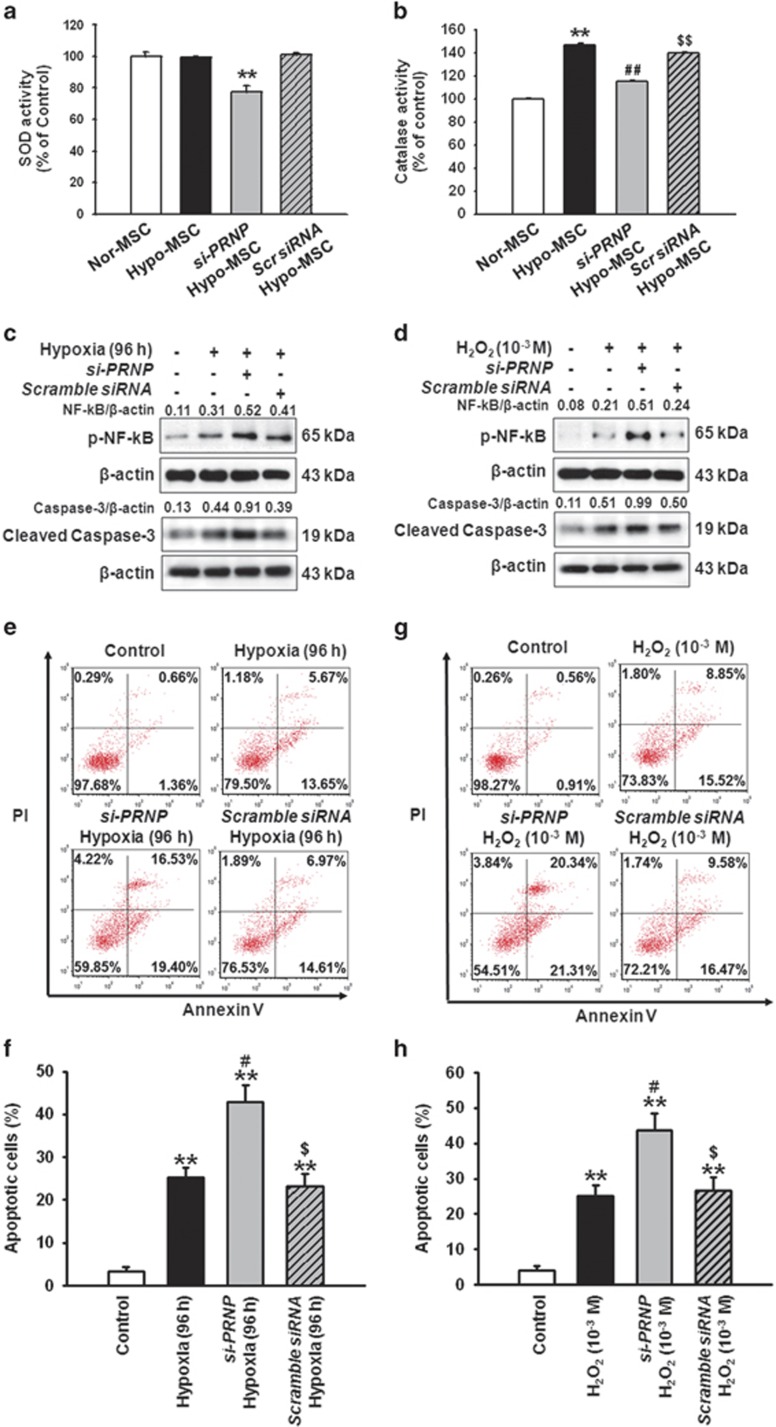
Knockdown of cellular prion protein (PrP^C^) expression results in reduced ROS-scavenging activity and increased oxidative stress-induced apoptosis. (**a** and **b**) After hypoxia preconditioning for 12 h, we evaluated the superoxide dismutase (SOD) and catalase activities of normoxic MSCs (Nor-MSC), hypoxia-preconditioned MSCs (Hypo-MSC), hypoxia-preconditioned MSCs transfected with PrP^C^-specific siRNA (si-PRNP Hypo-MSC), and hypoxia-preconditioned MSCs transfected with scrambled siRNA (Scr siRNA Hypo-MSC). Values represent means±S.E.M. **P<0.01 *versus* Nor-MSC; ^##^*P*<0.01 *versus* Hypo-MSC; ^$$^*P*<0.01 *versus* si-PRNP Hypo-MSC. (**c**) Western blot analysis of the levels of p-NF-*κ*B and cleaved caspase-3 in MSCs and MSCs pretreated with PrP^C^-specific siRNA or scrambled siRNA after exposure to hypoxic conditions for 96 h. (**d**) Western blot analysis of the levels of p-NF-*κ*B and cleaved caspase-3 in MSCs and MSCs pretreated with PrP^C^-specific siRNA or scrambled siRNA after exposure to H_2_O_2_ (10^−3^ M) for 3 h. (**e**) After exposure to hypoxia for 96 h, MSCs were treated with propidium iodide (PI) and an Annexin V stain, and the number of apoptotic cells was assessed by flow cytometry analysis. (**f**) Analysis of FACS experiments showing a significant apoptosis rate. **P<0.01 *versus* control; ^#^*P*<0.05 *versus* hypoxia (96 h); ^$^*P*<0.05 *versus* si-PRNP hypoxia (96 h). (**g**) After exposed to H_2_O_2_ (10^−3^ M) for 3 h, MSCs were treated with propidium iodide (PI) and an Annexin V stain, and the number of apoptotic cells was assessed by flow cytometry analysis. (**h**) Analysis of FACS experiments showing a significant apoptosis rate. **P<0.01 *versus* control; ^#^*P*<0.05 *versus* H_2_O_2_ (10^−3^ M); ^$^*P*<0.05 *versus* si-PRNP H_2_O_2_ (10^−3^ M)

To examine the effects of increased PrP^C^ expression on ROS-scavenging, the superoxide dismutase (SOD) and catalase activity of Hyp-MSCs were evaluated. Although hypoxia preconditioning had no significant effect on SOD activity, downregulation of PrP^C^ resulted in significantly decreased SOD activity in Hypo-MSCs compared with normoxic control group (Figure 3a). Conversely, MSCs subjected to hypoxic preconditioning exhibited significantly increased catalase activity, compared with that of MSCs cultivated under normoxic conditions, and this enhancement was blocked by treatment with the PrP^C^-specific siRNA (Figure 3b). To investigate whether PrP^C^ provides a protective effect against oxidative stress in MSCs, we assessed the levels of apoptosis in MSCs subjected to oxidative stress conditions, including long-term hypoxia (96 h) or treatment with H_2_O_2_ by western blot analysis and flow cytometry for detection of PI and Annexin V staining. In MSCs exposed to long-term hypoxia, siRNA-mediated downregulation of PrP^C^ resulted in increased levels of phosphorylated-nuclear factor kappa B (p-NF-*κ*B) and caspase-3 cleavage (Figure 3c and d), and increased numbers of apoptotic cells (Figure 3e and f). Similar results were observed in MSCs subjected to H_2_O_2_-induced oxidative stress conditions (Figure 3g and h). These data suggest that PrP^C^ protects MSCs against induction of oxidative stress-induced apoptosis through ROS-scavenging activity.

### Hypo-MSC yield improved functional recovery in a murine hindlimb ischemia model

**Figure 4:**
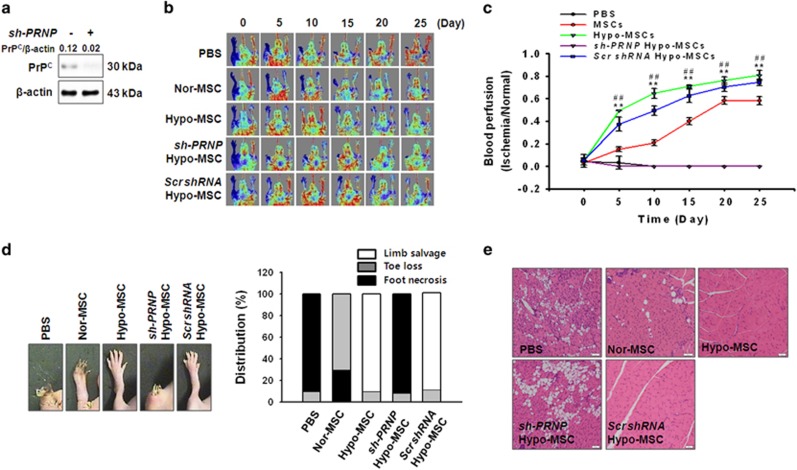
Assessment of functional recovery in a murine hindlimb ischemia model. (**a**) Western blot analysis of PrP^C^ expression in MSCs pretreated with PrP^C^-specific shRNA (sh-PRNP). (**b**) Blood flow recovery was evaluated via laser Doppler imaging analysis of the ischemic sites of mice injected with PBS, normoxic mesenchymal stem cells (Nor-MSC), hypoxia-preconditioned MSCs (Hypo-MSC), hypoxia-preconditioned MSCs transfected with PrP^C^-specific shRNA (sh-PRNP Hypo-MSCs), and hypoxia-preconditioned MSCs transfected with scrambled shRNA (Scr shRNA Hypo-MSCs) at 0, 5, 10, 15, 20, and 25 days post surgery. (**c**) The ratio of blood perfusion (blood flow in the left ischemic limb/blood flow in the right non-ischemic limb) was measured in each of the five treatment groups. Values represent means±S.E.M. **P<0.01 *versus* PBS; ^##^*P*<0.01 *versus* Nor-MSCs and sh-PRNP Hypo-MSCs. (**d**) Representative images illustrating the different outcomes (foot necrosis, toe loss, and limb salvage) observed for ischemic limbs in the five treatment groups at postoperative day 25. Distribution of the different outcomes in each group. (**e**) Necrosis of muscle tissues in the ischemic hindlimb was confirmed by hematoxylin and eosin staining. Scale bar=100 *μ*m

To determine whether MSCs subjected to hypoxia preconditioning exhibit enhanced vascular repair *in vivo*, blood perfusion and tissue repair were investigated following transplantation of PBS, Nor-MSCs, Hypo-MSCs, sh-PRNP Hypo-MSCs, and Scr shRNA Hypo-MSC into ischemic thigh muscles of murine hindlimbs. We used PRNP shRNA for stable PrP^C^ downregulation in MSCs. PrP^C^ expression was confirmed by western blotting (Figure 4a).

Blood perfusion was assessed by LDPI at postoperative days 0, 5, 10, 15, 20, and 25 (Figure 4b). Notably, mice receiving Hypo-MSCs exhibited significantly increased blood perfusion ratios than those in the PBS, Nor-MSC, and sh-PRNP Hypo-MSC groups (Figure 4c). Meanwhile, transplantation of Hypo-MSCs also resulted in significantly reduced levels of toe loss and foot necrosis compared with that observed in the other mouse groups (Figure 4d and e). These data indicate that transplantation of Hypo-MSC yield enhanced functional recovery in the murine hindlimb ischemia model through improvement of blood flow.

### Hypoxia preconditioning enhances MSC survival, proliferation, and neovascularization in ischemic tissues

**Figure 5:**
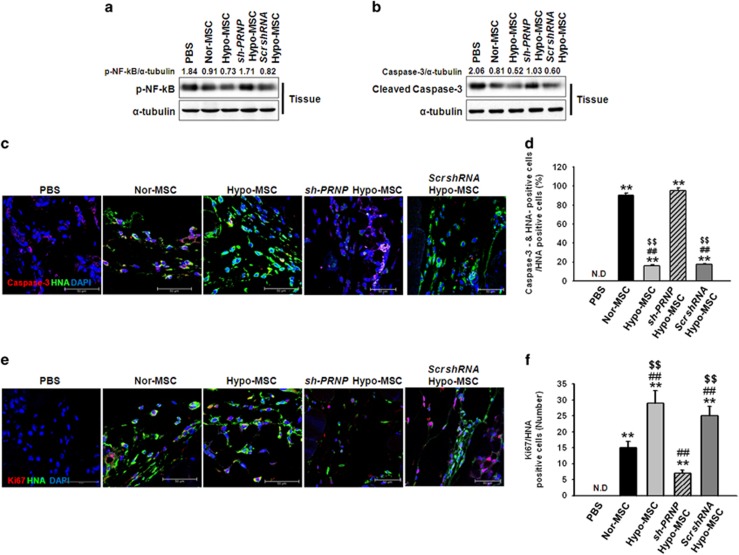
Hypoxia preconditioning enhances the survival and proliferation of transplanted mesenchymal stem cells (MSCs). (**a** and **b**) Western blot analysis of p-NF-*κ*B and cleaved caspase-3 expression in ischemic sites of mice injected with PBS, normoxic mesenchymal stem cells (Nor-MSC), hypoxia-preconditioned MSCs (Hypo-MSCs), hypoxia-preconditioned MSCs transfected with PrP^C^-specific shRNA (sh-PRNP Hypo-MSCs), and hypoxia-preconditioned MSCs transfected with scrambled shRNA (Scr shRNA Hypo-MSCs) at day 3. (**c**) The levels of MSC apoptosis were evaluated at 3 days after transplantation by immunostaining of ischemic limb tissues for human nuclear antigen (HNA) and cleaved caspase-3 expression. Scale bar=50 *μ*m. (**d**) The levels of apoptosis of transplanted MSCs were quantified as the HNA and cleaved caspase-3 double-positive cells/HNA-positive cells. Values represent the mean±S.E.M. **P<0.01 *versus* PBS; ^##^*P*<0.01 *versus* Nor-MSC; ^$$^*P*<0.01 *versus* sh-PRNP Hypo-MSCs. (**e**) MSC proliferation was evaluated by immunostaining for HNA and Ki67 expression in ischemic limb tissues at 3 days after hMSC transplantation. Scale bar=50 *μ*m. (**f**) Proliferation of transplanted MSCs was quantified as the number of HNA and Ki67 double-positive cells. Values represent the mean±S.E.M. **P<0.01 *versus* PBS; ^##^*P*<0.01 *versus* Nor-MSC; ^$$^*P*<0.01 *versus* sh-PRNP Hypo-MSCs

**Figure 6:**
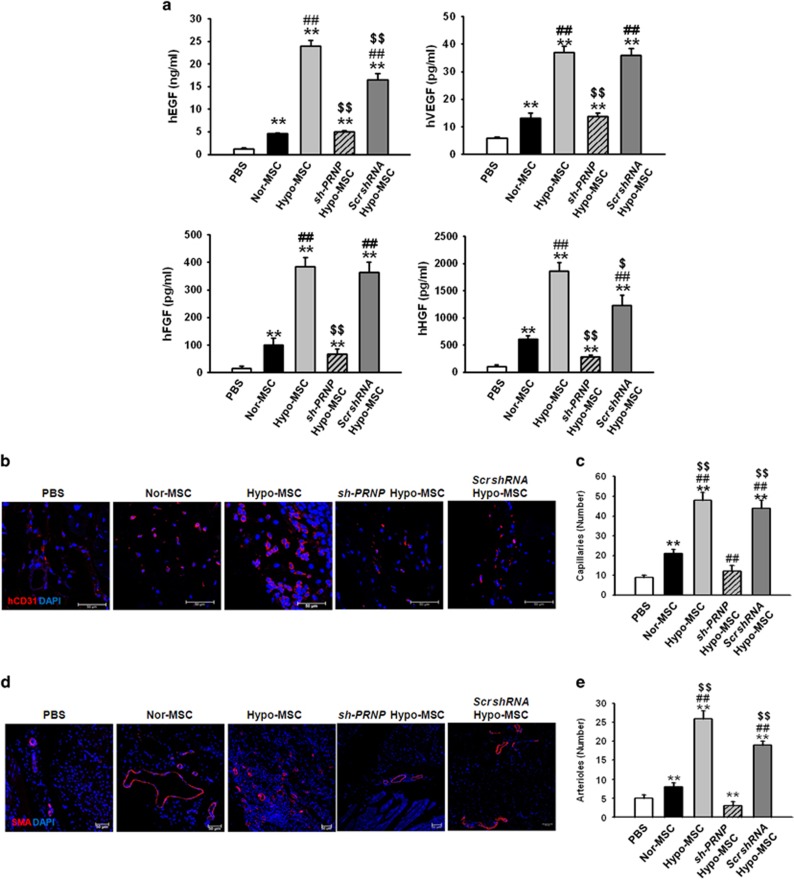
Hypoxia-preconditioned mesenchymal stem cells (Hypo-hMSCs) promote enhanced neovascularization in a murine hindlimb ischemia model. (**a**) The expression of hEGF, hVEGF, hFGF, and hHGF in ischemic limb tissue lysates was determined by ELISA. Values represent the mean±S.E.M. **P<0.01 *versus* PBS; ^##^*P*<0.01 *versus* Nor-MSCs; ^$^*P*<0.05 *versus* sh-PRNP Hypo-MSCs ^$$^*P*<0.01 *versus* sh-PRNP Hypo-MSCs. (**b**) Capillary density was evaluated in ischemic tissues at 25 days post MSC transplantation by immunostaining for CD31. Scale bar=50 *μ*m. (**c**) Capillary density was quantified as the number of human CD31-positive cells. Values represent the mean±S.E.M. **P<0.01 *versus* PBS; ^##^*P*<0.01 *versus* Nor-MSC; ^$$^*P*<0.01 *versus* sh-PRNP Hypo-MSC. (**d**) Arteriole density in ischemic tissues was evaluated at 25 days post MSC transplantation by immunostaining with an *α*-SMA antibody. Scale bar=50 *μ*m. (**e**) Arteriole density was quantified as the number of *α*-SMA-positive cells. Values represent the mean±S.E.M. **P<0.01 *versus* PBS; ^##^*P*<0.01 *versus* Nor-MSC; ^$$^*P*<0.01 *versus* sh-PRNP Hypo-MSCs

To explore whether hypoxia preconditioning augments the survival and proliferation of transplanted MSCs, ischemic tissues were harvested at postoperative day 3 and subjected to western blot analysis for p-NF-*κ*B and cleaved caspase-3, and immunofluorescence staining for HNA, cleaved caspase-3, and Ki67 (Figure 5). The phosphorylation of NF-*κ*B and expression of cleaved caspase-3 was significantly decreased in tissue lysates injected with hypo-MSCs compared within other groups (Figure 5a and b). The levels of apoptosis among the MSCs in the tissues of each group were then assessed by quantifying the number of HNA and cleaved caspase-3 double-positive cells (Figure 5c and d), whereas the levels of MSC proliferation were evaluated by quantifying the number of HNA and Ki67 double-positive cells (Figure 5e and f). There were significantly fewer apoptotic MSCs within ischemic sites transplanted with Hypo-MSCs than in those transplanted with Nor-MSCs or sh-PRNP Hypo-MSCs (Figure 5d). Meanwhile, ischemic tissues transplanted with Hypo-MSCs contained greater numbers of proliferating MSCs than those transplanted with Nor-MSCs or sh-PRNP Hypo-MSCs (Figure 5f). To confirm angiogenic cytokine expression after the transplantation of MSCs, we assessed the expression of human epidermal growth factor (hEGF), human vascular endothelial growth factor (hVEGF), human fibroblast growth factor (hFGF), and human hepatocyte growth factor (hHGF) at postoperative day 3. The expression levels of hEGF, hVEGF, hFGF, and hHGF were significantly higher in ischemic tissue injected with Hypo-MSCs than in tissues from other groups (Figure 6a). To evaluate the effects of Hypo-MSC on neovascularization, we quantified the number of CD31-positive capillaries and *α*-SMA-positive arterioles within the ischemic tissues of mice in each group at postoperative day 28 by immunofluorescence staining. There was a significantly greater density of capillaries and arterioles within the ischemic tissues of mice transplanted with Hypo-MSCs than in those transplanted with Nor-MSCs or sh-PRNP Hypo-MSCs (Figure 6b–e). These findings therefore imply that hypoxia preconditioning promotes the survival and proliferation of transplanted MSCs in ischemic tissues *in vivo*, thereby yielding increased transplanted MSC-mediated neovascularization.

## Discussion

MSCs have important roles in the maintenance and repair of bone and blood tissues. In principle, MSCs can be readily isolated from patient tissues, including bone marrow and adipose tissues, and then expanded in culture until the large numbers required for clinical application are obtained. However, the application of MSCs for stem cell-based therapies is hindered by several limitations, including low proliferative potential, restricted life span, and gradual loss of ‘stemness' during *ex vivo* expansion.^20, 21, 22^ In addition, exposure to stressful conditions such as oxidative stress, ischemia, and inflammation can induce apoptosis and cell death, which has resulted in low efficacies of MSCs transplanted into injured tissues.^20, 23, 24, 25^ Notably, although the tissues from which MSCs are isolated are associated with variable oxygen levels (1–7 % in bone marrow, 10–15% in adipose tissue, and 2–8% at the MSC niche),^26, 27^ MSCs are typically cultured under normoxic conditions, resulting in the induction of oxidative stress and the alteration of DNA, biomolecule structures, and metabolism.^28^ To address this issue, several studies have demonstrated that hypoxia preconditioning of MSCs results in reduced apoptosis, chromosomal abnormality, and senescence, and enhanced paracrine activity and regenerative potential.^20, 28, 29^

Although there is increasing evidence highlighting the beneficial effects of hypoxia preconditioning on MSCs, the precise mechanism through which preconditioning affects these cells remains unclear. Likewise, although there is a consensus that PrP^C^ enhances stem cell proliferation *in vitro* and *in vivo*,^30^ the molecular mechanism through which PrP^C^ influences the proliferative potential of MSCs remains unclear. Here, we provide the first evidence that hypoxia treatment results in enhanced expression of PrP^C^, which in turn promotes MSC expansion via activation of the JAK2/STAT3 pathway. Previous reports revealed that STAT3 contributes to hypoxia-induced enhancement of self-renewal in embryonic and other adult stem cells.^31, 32, 33^ Furthermore, our previous study indicated that hypoxia increases the proliferation of endothelial colony-forming cells through the activation of JAK2/STAT3 pathway.^19^ These findings suggest that hypoxia-induced PrP^C^ enhances MSC proliferation via JAK2/STAT3 pathway activation, and that PrP^C^ is key regulator of JAK2/STAT3 signaling in MSCs.

Importantly, we also demonstrated that maintenance of PrP^C^ expression protected MSCs against oxidative stress-induced apoptosis by promoting increased SOD and catalase activity. Our data are therefore in agreement with previous studies showing that PrP^C^ confers enhanced resistance to oxidative stress by contributing to cellular SOD and catalase activity, whereas attenuated PrP^C^ expression results in enhanced sensitivity to oxidative stress.^34, 35, 36^ Interestingly, PrP^C^ has a pro-apoptotic role during ER stress, but an anti-apoptotic role during oxidative stress-induced cell death.^37^ Our data indicate that hypoxia preconditioning-mediated PrP^C^ expression prevents oxidative stress-induced apoptosis and cell death through the regulation of SOD and catalase activity.

Although MSCs differentiate into smooth muscle, endothelial cells, or myocardial muscle *in vivo*, the predominant mechanism of MSC-mediated tissue repair occurs through angiogenic factor secretion. Accumulating evidence suggests that hypoxia enhances the secretion of VEGF, IGF-1, SDF-1, and EPO, indicating that hypoxic preconditioning improves the tissue repair potential of MSCs.^38, 39, 40^ However, successful cell engraftment is determined by several factors, including the rates of cell apoptosis and death, the extent of mechanical loss, and the rates at which the cells escape from the region of transplantation, influence the rates of cell engraftment and proliferation in ischemic sites.^1^ Our current study shows that hypoxia preconditioning yields enhanced functional recovery in a murine hindlimb ischemia model via augmentation of survival, proliferation, and neovascularization of transplanted MSCs. Moreover, the beneficial effects of hypoxia preconditioning on tissue repair and vascularization appears to be mediated by regulation of PrP^C^ expression. Consistent with these findings, overexpression of PrP^C^ was shown to provide a protective effect against focal cerebral ischemia via the activation of ERK, whereas downregulation of PrP^C^ increased the exacerbation of ischemic brain injury through post-ischemic activation of caspase-3.^41, 42^ Moreover, PrP^C^ was found to promote neuroprotection, neurogenesis, and angiogenesis in ischemic brain injuries.^18^

## Conclusion

In this study, we investigated the efficacy of *ex vivo* hypoxic preconditioning for enhancing the proliferative potential of MSCs, as well as their resistance to ischemic stress-induced apoptosis. The optimized culture conditions examined here promoted robust cell survival, proliferation, and protection against oxidative stress, resulting in improved therapeutic efficacy of MSCs in a murine hindlimb ischemia model, suggesting that hypoxic preconditioning could comprise a new therapeutic strategy for overcoming ischemic diseases. In addition, our results indicate that PrP^C^ might represent a novel target for MSC-based therapies for several ischemic diseases.

## Materials and Methods

### Cell cultures

Human adipose-derived MSCs were obtained from the American Type Culture Collection (ATCC; Manassas, VA, USA) and were pathogen- (HBV, HCV, HIV, and Syphilis) and mycoplasma-negative. The supplier certified that the MSCs exhibited expression of the cell surface markers CD73 and CD105, but not of CD31, as well as adipogenic and osteogenic differentiation potential when cultured in specific differentiation media. Cells were cultured in alpha-minimum essential medium (Thermo-Fisher Scientific, Waltham, MA, USA) supplemented with 10% (v/v) fetal bovine serum (Thermo-Fisher Scientific), 100 U/ml of penicillin, and 100 mg/ml streptomycin. Cells were growing a humidified incubator at 37 °C with 5% CO_2_.

### MSC differentiation

For MSC differentiation, cells were grown in StemPro adipogenic, osteogenic, or chondrogenic culture medium (Thermo-Fisher Scientific). To detect adipocytes, cells were cultivated in adipogenic medium for 21 days and stained with oil red O (Sigma-Aldrich, St. Louis, MO, USA) for 15 min. To detect osteoblasts, cells were grown in osteogenic medium for 21 days and subjected to alkaline phosphatase staining with an alkaline phosphatase kit (Sigma-Aldrich). Chondrocytes were detected by staining cells grown in chondrogenic medium for 14 days with safranin O (Sigma-Aldrich).

### Quantitative real-time PCR

qPCR analysis was performed using the Rotor-Gene 6000 real-time thermal cycling system (Corbett Research, Mortlake, NSW, Australia) with a QuantiMix SYBR Kit (Phile Korea Technology, Daejeon, Korea). PCR was performed under the following cycling conditions: 95 °C for 15 s, annealing at 60 °C for 30 s, and extension at 72 °C for 60 s for 40 cycles. The data were analyzed by the comparative threshold cycle (CT) method and normalized against Gapdh controls. Primer sequences were as follows: Gapdh forward, 5′-AGTATGACTCCACTCACGGCAA-3′ Gapdh reverse 5′-TCTCGCTCCTGGAAGATGGT-3′ fabp4 forward, 5′-TCGACTTTCCATCCCACTTC-3′ fabp4 reverse, 5′-AGTGACCTCTGTTCGAAGGT-3′ Osteopontin forward, 5′-ATGAGAGCCCTCAGACTCCTC-3′ Osteopontin reverse, 5′-CGCGCCGTAGAAGCGCCGATA-3′, Sox9 forward, 5′-CTGAACGAGAGCGAGAAG-3′ Sox9 reverse, 5′-TTCTTCACCGACTTCCTCC-3′.

### Single-cell cultivation assay

A limited-dilution assay was used to aliquot single MSCs into individual wells of 96-well culture plates. In brief, cell suspensions containing 1 × 10^3^ cells in 10 ml complete medium were diluted 1:10 (cells: complete medium), and 100 *μ*l of the diluted sample (approximately 1 cell/100 *μ*l) was seeded into 96-well plates. Cells were then cultured under normoxic or hypoxic conditions in a humidified incubator, and each well was examined for MSC grow that day 10.

### Hypoxia preconditioning

MSCs were incubated in a modular incubator chamber (IB Science, Daejeon, Korea) maintaining a hypoxic gas mixture composed of a 2% O_2_, 5% CO_2_, and balanced N_2_ for 0, 6, 12, and 24 h at 37 °C.

### siRNA transfection

hMSCs were grown to 70% confluence in culture dishes and were transfected for 48 h with SMART pool siRNAs (100 nM/l) specific to HIF-1*α* or PrP^C^ mRNA using Lipofectamine 2000 reagent (Thermo-Fisher Scientific), according to the manufacturer's protocols.

### Western blot analysis

Cells and tissue homogenates (20 *μ*g protein) were separated via 10% sodium dodecyl sulfate-polyacrylamide gel electrophoresis and transferred to nitrocellulose membranes for antibody probing. After washing with TBST (10 mM Tris–HCl (pH 7.6), 150 mM NaCl, 0.05% Tween-20), membranes were blocked with 5% skim milk for 2 h and then incubated with primary antibodies specific to HIF-1*α*, PrP^C^, janus kinase 2 (JAK2), phosphorylated-JAK2, signal transducer and activator of transcription 3 (STAT3), p-STAT3, cyclin D1, c-Myc, p-NF-*κ*B, cleaved caspase-3, and *β*-actin (Santa Cruz Biotechnology, Dallas, TX, USA). After incubation of the membranes with peroxidase-conjugated secondary antibodies (Santa Cruz Biotechnology), bands were detected using enhanced chemiluminescence reagents (Amersham Biosciences, Little Chalfont, UK) in a dark room.

### SOD activity assay

The protein concentration of cells lysate was determined using bicinchoninic acid assay (Sigma). The SOD activity assay is based on water soluble tetrazolium reduction by superoxide anion (produced from xanthine by xanthine oxidase) to a colored water soluble tetrazolium formazan product and was performed the total SOD activity measurement at 480 nm.

### Catalase activity assay

Catalase activity was based on the rate of degradation of H_2_O_2_.The cells were removed from the culture dish by scraping with a policeman, and cells lysate protein extracts (40 *μ*g) reaction sample contained the indicated quantities of catalase and 20 mM H_2_O_2_ in 0.1 M Tris–HCl, and incubated for 30 min, and 50 mM Amplex red reagent and 0.2 U/ml of horseradish peroxidase was added and incubated for 30 min at 37 °C. The decomposition of the substrate was recorded by the decrease in absorbance at 563 nm.

### PI/Annexin V flow cytometry analysis

To examine the levels of cell apoptosis, cells were stained with Annexin V-FITC and propidium iodide (PI) (*De Novo* Software, Los Angeles, CA, USA) and evaluated using a Cyflow Cube 8 kit (Partec, Münster, Germany). Data were analyzed using standard FSC Express software (*De Novo* Software).

### PrP^C^ knockdown by shRNA

The lentiviral PRNP shRNA constructs were obtained from OriGene Technologies (Rockville, MD, USA). At 24 h after cell seeding, 293T cells (1.5 × 10^6^) were transfected with a lentiviral vector encoding PRNP shRNA (3 *μ*g) and packaging vector pGFP-C-shLenti (0.3 *μ*g; OriGene Technologies) using Lipofectamine 2000 (Invitrogen, Carlsbad, CA, USA). Forty-eight hours after transfection, the supernatant harboring lentiviruses was collected and filtered using a sterilized 0.45-*μ*m syringe filter. The PRNP shRNA lentiviral supernatant with polybrene (5 *μ*g/ml) was added to the hMSCs for infection. After selection in growth medium containing puromycin (2 *μ*g/ml) for 2 weeks, individual resistant cells were expanded in growth media without puromycin, and protein expression was evaluated via western blot analysis. PRNP shRNA No. 3 showed the most effective PrP^C^ knockdown among the four PRNP shRNA clones (No.1–4). The corresponding knockdown clone was used for further studies. The PRNP shRNA No. 3 sequence was 5′-AACCAACATGAAGCACATGGCTGGTGCTG-3′.

### Cell transplantation in a murine hindlimb ischemia model

Cell transplantation experiments were performed on 8-week-old nude male BALB/c mice (Biogenomics, Seoul, Korea) maintained under a 12-h light/dark cycle, and in accordance with the regulations of Soonchunhyang University, Seoul Hospital. All animal procedures were approved by the Institutional Animal Care and Use Committee of Soonchunhyang University, Seoul Hospital, Korea (IACUC2013-5). To induce ischemia and oxidative stress and to assess neovascularization, we utilized a hindlimb ischemia model, as previously described but with minor modifications.^43, 44^ Ischemia was induced by ligation of the proximal femoral artery and the boundary vessels of the mice. At no later than 6 h after surgery, PBS, Nor-MSCs, Hypo-MSC, Hypo-MSC transfected with PrP^C^-specific shRNA (sh-PRNP Hypo-MSCs), or Hypo-MSC transfected with scrambled shRNA (Scr shRNA Hypo-MSCs) were injected intramuscularly into the ischemic thigh area (5 × 10^5^ cells/100 *μ*l PBS per mouse; five mice per treatment group). Cells were injected into five distinct ischemic sites. Blood perfusion was assessed by measuring and comparing the ratio of blood flow in the ischemic (left) limb to that in the non-ischemic (right) limb via laser Doppler perfusion imaging analysis (Moor Instruments, Wilmington, DE, USA) on postoperative days 0, 5, 10, 15, 20, and 25.

### Determination of human growth factors

The levels of hEGF, hVEGF, hFGF, and hHGF in the ischemic limb tissue (at 3 days post surgery) lysates were determined by ELISA using a commercially available ELISA kit (R&D Systems, Minneapolis, MN, USA) according to the manufacturer's recommendation. All proteins were quantified using a bicinchoninic acid assay (BCA protein assay; Thermo Scientific, Waltham, MA, USA). Growth factor expression levels were quantified by measuring the absorbance at 450 nm using a microplate reader (Tecan Group AG, Männedorf, Switzerland).

### Immunohistochemistry

Ischemic thigh areas were removed at 3 or 25 days post MSC transplantation, fixed with 4% paraformaldehyde (Affymetrix, Santa Clara, CA, USA), embedded in paraffin, and sectioned. For histological analyses, sections were subjected to hematoxylin and eosin staining, followed by immunofluorescence staining in a dark room with primary antibodies against CD31, alpha-smooth muscle actin (*α*-SMA), cleaved caspase-3, Ki67 (Santa Cruz Biotechnology) or human nuclear antigen (HNA; Millipore, Billerica, MA, USA), and secondary antibodies conjugated to Alexa488 or Alexa594 (Thermo-Fisher Scientific). Nuclei were visualized by staining with 4′,6-diaminido-2-phenylindol (DAPI; Sigma-Aldrich). Immunostained slides were imaged by confocal microscopy (Olympus, Tokyo, Japan).

### Statistical analyses

All data are presented as means±S.E.M. All experimental results were evaluated by analysis of variance, followed by a comparison of the treatment and control groups using the Bonferroni–Dunn test. *P*-values<0.05 were considered statistically significant.

## Abbreviations

MSCs: mesenchymal stem cells
PrP^C^: cellular prion protein
ROS: reactive oxygen species
HIF-1*α*: hypoxia-inducible factor-1 alpha
CXCR4: C-X-C chemokine receptor type 4
JAK2: janus kinase 2
STAT3: signal transducer and activator of transcription 3
NF-*κ*B: nuclear factor kappa B
SOD: superoxide dismutase
hEGF: human epidermal growth factor
hVEGF: human vascular endothelial growth factor
hFGF: human fibroblast growth factor
hHGF: human hepatocyte growth factor
IGF-1: insulin-like growth factor-1
SDF-1: stromal cell-derived factor-1
EPO: erythropoietin
*α*-SMA: alpha-smooth muscle actin
HNA: human nuclear antigen
DAPI: 4′,6-diaminido-2-phenylindol
FABP4: fatty acid-binding protein 4
GFP: green fluorescent protein
ERK: extracellular signal-regulated protein kinase
H_2_O_2_: hydrogen peroxide
ECL: enhanced chemiluminescence
HCl: hydrochloric acid
PI: propidium iodide
FITC: fluorescein isothiocyanate
